# Identification of the *Dof* Gene Family in Quinoa and Its Potential Role in Regulating Flavonoid Synthesis Under Different Stress Conditions

**DOI:** 10.3390/biology14040446

**Published:** 2025-04-20

**Authors:** Guangtao Qian, Jinrong Yang, Mingyu Wang, Lixin Li

**Affiliations:** 1 Interdisciplinary Eye Research Institute (EYE-X Institute), Bengbu Medical University, Bengbu 233030, China; qgt@bbmu.edu.cn (G.Q.); 15156172852@163.com (J.Y.); 2 Anhui Provincial Key Laboratory of Tumor Evolution and Intelligent Diagnosis and Treatment, School of Life Sciences, Bengbu Medical University, Bengbu 233030, China; 3 Key Laboratory of Saline-Alkali Vegetation Ecology Restoration, Ministry of Education, School of Life Sciences, Northeast Forestry University, Harbin 150040, China; wmy19970825@163.com

**Keywords:** Dof transcription factor, quinoa, light quality, flavonoid, abiotic stress

## Abstract

Quinoa (*Chenopodium quinoa* Willd.), often referred to as the “golden grain”, is a highly nutritious crop that has garnered significant global attention due to its exceptional nutritional profile and health benefits. Flavonoids present in quinoa have been shown to possess antioxidant, anti-inflammatory, antiviral, anticancer, and antidepressant properties. The DNA binding with one finger (Dof) transcription factor is crucial for regulating growth, development, and stress responses. However, the identification of the Dof family using the latest quinoa genomic data and its function in abiotic stress response have not been fully elucidated. Here, 36 *CqDof* genes were identified from the quinoa genome and classified into ten subfamilies through phylogenetic analysis. Physicochemical property analysis predicted that *CqDofs* predominantly encode basic, hydrophilic, and unstable nuclear proteins. *CqDofs* were distributed across 15 chromosomes, with segmental duplication being the primary driver of their expansion. Subsequently, basic information on *CqDofs* was systematically analyzed, including conserved motifs, gene structure, cis-acting elements, and expression patterns. Notably, the promoter regions of all *CqDof* genes were enriched with cis-acting elements related to light responsiveness. Further analysis revealed that red and blue light significantly affected *CqDof* expression and flavonoid accumulation (epigallocatechin, rutin, naringenin, morin, pinocembrin, quercetin-7-O-rutinoside, quercetin-3-O-glucoside, and naringenin), in which 5 *CqDofs* exhibited a pronounced response to both light conditions and showed a significant correlation with flavonoid levels. Finally, RT-PCR analysis indicated that the expression levels of *CqDofs* (except *CqDof21*) were significantly upregulated under drought, salt, and saline-alkali stresses. These findings lay the groundwork for future studies on how *CqDofs* regulate flavonoid biosynthesis under different light qualities and function in abiotic stress.

## Simple Summary

This study examines the *Dof* gene family in quinoa, focusing on its role in flavonoid synthesis under different light conditions. A total of 36 *CqDof* genes were identified and divided into 10 subfamilies. These genes encode basic, hydrophilic, unstable nuclear proteins and are distributed across 15 chromosomes, with segmental duplication driving their expansion. Their promoters contain elements related to light responsiveness. Red and blue light significantly influences the expression of *CqDofs* and flavonoid accumulation, with 5 *CqDofs* showing a strong response and correlation with flavonoid levels. RT-PCR analysis also shows that *CqDof* expression is upregulated under drought, salt, and saline-alkali stress, except for *CqDof21*. These findings lay the groundwork for future research on the regulatory mechanisms of *CqDofs* in flavonoid biosynthesis under varying light qualities and their functions under abiotic stress.

## 1. Introduction

The dicotyledonous plant quinoa (*Chenopodium quinoa* Willd.), a member of the Amaranthaceae family, has been cultivated for nearly 7000 years [1]. Quinoa is known for its high nutritional value, including a wide range of proteins, amino acids, vitamins, minerals, unsaturated fatty acids, and dietary fiber [2]. Remarkably, quinoa seeds provide all essential amino acids required for human nutrition [3]. Furthermore, quinoa contains bioactive substances such as flavonoids, phenolic acids, and terpenoids, which possess antioxidant, anti-inflammatory, antimicrobial, and anti-hypertensive properties, contributing to the prevention and treatment of cancer, heart disease, obesity, and neurodegenerative illnesses [4]. These attributes have led to a significant increase in quinoa production and consumption in recent years.

Plants are subjected to a variety of abiotic and biotic stresses during growth and development, including light, temperature, salinity, and heat [5]. Therefore, plants have evolved diverse adaptive strategies and signaling mechanisms to deal with these stresses. Transcription factors (TFs), including members of the NAC, MYB, bHLH, AP2/ERF, and *Dof* families, play crucial roles in regulating plant stress responses and enhancing plant resilience in these stressful environments [6]. Quinoa is a uniquely stress-tolerant crop and one of the most significant food crops globally, but these stresses also limit yield and quality [7]. Given its importance, quinoa has been extensively studied for stress tolerance, particularly to heat, drought, and cold. For example, CqERF24 enhanced drought tolerance by increasing antioxidant enzyme activities and activating stress-responsive genes [8], while CqZF-HD14 did so by interacting with CqHIPP34 and CqNAC79 [9]. Furthermore, heat stress changed fatty acid and mineral nutrient concentrations in quinoa seeds [10], and heat stress factors (HSFs) such as CqHsfs10 and CqHsfs4 are critical in quinoa thermotolerance [11]. As a facultative halophyte, quinoa exhibits remarkable salt tolerance, with some varieties enduring up to 400 mM NaCl [12]. However, salt stress can disrupt metabolism and inhibit growth. Light significantly impacts plant growth, development, and secondary metabolite biosynthesis, including flavonoids, which are renowned for their antioxidant properties and health benefits [13]. It should be highlighted that light quality is species-specific in regulating the accumulation of flavonoids. For instance, *Camellia sinensis* could accumulate higher levels of flavonoids when exposed to blue light [14], while red light significantly promoted flavonoid levels in *Scrophularia kakudensis* [15]. In quinoa, UV-B irradiation influences physiological responses and flavonoid accumulation, with studies showing a reduced total flavonoid content but an increased antioxidant capacity under UV-B treatment [16]. Additionally, UV-B intensity and duration affect chlorophyll and carotenoid levels in quinoa [17]. Overall, research on quinoa has concentrated on the impact of UV-B treatment, but the influence of monochromatic light, specifically blue and red light, on the synthesis of important flavonoids in quinoa remains largely unexplored.

The Dof (DNA binding with one finger) transcription factor, a member of the zinc finger superfamily, typically consists of 200–400 amino acids and features a C2–C2 type single zinc finger domain with 50–52 conserved residues, including one Zn^2+^ and four conserved Cys residues [18]. Furthermore, Dof TFs bind specifically to DNA sequences containing a 5′-AAAG-3′ core in target gene promoters [19]. Currently, numerous Dof TF members have been identified in various plants, including *Oryza sativa*, *Gossypium hirsutum*, *Medicago polymorpha*, *Arabidopsis thaliana*, and *Brassica napus*, with counts of 30, 51, 36, 36, and 117 *Dof* members, respectively [6]. Recent studies highlight the significance of Dof TFs in plant growth, development, stress responses, and secondary metabolite biosynthesis. For example, COG1 promotes photosynthesis and starch accumulation, affecting plant biomass [20]. Additionally, VDOF1 and VDOF2 can be involved in the regulation of vascular cell differentiation and lignin biosynthesis in *Arabidopsis* [21]. Overexpression of *GhDof1.7* significantly increased salt tolerance in *Arabidopsis*, while silencing this gene reduced salt tolerance in cotton [22].

The quinoa genome was successfully assembled in 2017, laying the groundwork for comprehending its unique nutritional properties, stress tolerance mechanisms, and molecular breeding [23]. Subsequently, Rey et al. performed a chromosome-scale assembly of the Chilean accession PI 614886 (QQ74) using in vitro and in vivo Hi-C data [24]. Although recent quinoa genome data have provided abundant information, the function of *Dof* genes in quinoa’s response to abiotic stresses remains to be fully explored. Therefore, this study identified *Dof* family members in quinoa and analyzed their protein physicochemical properties, chromosomal locations, motif composition, gene structures, conserved domains, and cis-acting elements. Additionally, we investigated flavonoid content changes under red and blue light and correlated these with *CqDof* expression. Finally, five *CqDof* genes responsive to both red and blue light were selected and examined for their expression patterns under abiotic stress. These findings lay the groundwork for future investigations into their molecular response mechanisms under different light treatments and abiotic stresses and provide candidate genes for breeding strong stress-resistant quinoa in the future.

## 2. Materials and Methods

### 2.1. Plant Material and Abiotic Stress Treatments

The Jiaqi 3 quinoa variety (seed color: white; primary origin: Bolivia; ecotype: Altipolano) was obtained from Jiaqi Agricultural Technology Co., Ltd. (Taiyuan, China); it was used in our previous study and has a certain tolerance to saline-alkali stress [25]. Quinoa seeds were cultivated in a greenhouse at 25 °C under long-day conditions (16 h light/8 h dark, cool white fluorescent light) with a commercial substrate (Pindstrup Mosebrug A/S, Ryomgaard, Denmark). After 20 days of growth, quinoa seedlings were subjected to different abiotic treatments. Specifically, the seedlings were treated with salt (200 mM NaCl) [26], drought (30% PEG6000) [27], saline-alkali stress (100 mM Na_2_CO_3_:NaHCO_3_ = 1:9, molar ratio) [25], and monochromatic spectral light-emitting diodes (LEDs) emitting the following wavelengths: blue light (470 nm, 50 ± 5 μmol⋅m^−2^⋅s^−1^) and red light (670 nm, 50 ± 5 μmol⋅m^−2^⋅s^−1^) [28]. Quinoa seedling leaves were collected after 48 h of abiotic stress, while samples from the light treatment were harvested after 7 days. Thirty quinoa seedlings were collected from each treatment, randomly divided into 3 groups, and the leaves were immediately frozen in liquid nitrogen and stored at −80 °C for later analysis.

### 2.2. Identification and Basic Information Analysis of CqDofs

The genome sequences of quinoa, *A. thaliana*, and *O. sativa* were obtained from the NCBI database (https://www.ncbi.nlm.nih.gov, accessed on 15 September 2024). The *A. thaliana* Dof protein (*AtDof*) sequences were downloaded from the TAIR database (https://www.arabidopsis.org/, accessed on 16 September 2024). Additionally, the Dof domain was obtained from the Pfam database (http://pfam.xfam.org/family/PF02701, accessed on 16 September 2024). All Dof protein sequences from *Arabidopsis* were utilized as the query for BLASTp (E-value ≤ 1 × 10^−5^) to identify potential *Dof* family members in quinoa. The Pfam database (PF02701) was used to further screen the conserved domains of the candidate genes, and after removing incomplete domains and redundant sequences, the *CqDof* members were obtained. The fundamental features of *CqDofs*, including amino acid length, molecular weight (MW), theoretical isoelectric point (PI), instability index, grand average hydropathicity (GRAVY), and aliphatic index, were analyzed using online program ExPASy (https://www.expasy.org/, accessed on 20 September 2024). Subcellular localization prediction analysis of *CqDofs* was conducted using WoLF PSORT web tool (https://wolfpsort.hgc.jp/, accessed on 20 September 2024).

### 2.3. Multiple Sequence Alignment, Phylogenetic Relationship, and Conserved Domain Analysis of CqDofs

The CqDof and AtDof protein sequences were first aligned using ClustalW with default parameters. Subsequently, a phylogenetic analysis was performed using the neighbor-joining (NJ) method in MEGA11.0 software with 1000 bootstrap replicates, while adhering to the default settings for other parameters [6]. Ultimately, the evolutionary tree was visualized with the iTOL web tool (https://itol.embl.de/, accessed on 28 September 2024) for better visualization. The CqDof protein sequences were submitted to the NCBI-CDD database for structural domain analysis, and the results were visualized using TBtools (Version V2.154).

### 2.4. Chromosomal Localization, Gene Duplication Events, and Collinearity Analysis of CqDofs

According to the GFF annotation file of the quinoa genome [24], the number, length, starting and ending positions of chromosomes in quinoa were obtained, and the locations of *CqDofs* on chromosomes were visualized using TBtools software (Version V2.154). Duplication events of *CqDofs* and synteny to different species (*Arabidopsis thaliana*, *Oryza sativa*) were analyzed using MCScanX and plotted using TBtools software with default parameters [6].

### 2.5. Gene Structure, cis-Acting Element, and Gene Expression Analysis of CqDofs

The TBtools software visualized the number and location of the untranslated region (UTR) and coding sequence (CDS) in *CqDofs* based on the quinoa genome structure annotation information. The conserved motifs of CqDof proteins were analyzed using MEME (https://meme-suite.org/meme/tools/meme, accessed on 18 October 2024), with the motif parameter set to 15. The 2000 bp upstream sequences located immediately before the initiation codon of *CqDofs* were extracted. PlantCare (http://www.plantcare.co.uk/, accessed on 22 October 2024) was then employed to analyze and predict the cis-acting elements within this sequence. Furthermore, RNA-seq data from various tissues and organs (root, stem, leaf, flower, and fruit, accession numbers: PRJNA658178 and PRJNA578698) of quinoa, as well as data from salt stress of quinoa seedlings (accession numbers: PRJNA636120) were downloaded from NCBI. The fragments per kilobase of exon model per million mapped fragments (FPKM) values for each RNA-seq dataset were extracted, and the data of *CqDofs* were normalized using the log2 FPKM approach. All results were visualized using TBtools software with the default program.

### 2.6. RT-PCR Analysis of CqDofs Under Abiotic Stress

Total RNA and cDNA from each sample were extracted and reverse-transcribed using an RNA Extraction Kit (Tiangen Biotech, Beijing, China) and a SuperScript III reverse transcription kit (Tiangen Biotech, Beijing, China), respectively, following the manufacturer’s instructions. RT-PCR assays were performed on a Rotor-Gene Q system (Qiagen, Hilden, Germany) with a program that was consistent with that previously described [28]. Primer Premier 6.0 software was used to design RT-PCR primers, with *UBQ9* (AUR62020068) as an internal control [29]. All RT-PCR primers are listed in Table S1. The RT-PCR contained three biological replicates, and the relative expression level of the target genes was determined using the 2^−ΔΔCT^ method.

### 2.7. Sample Preparation and LC–MS Analysis

The light-treated quinoa leaves were freeze-dried for 24 h, followed by being completely ground with liquid nitrogen. Subsequently, 100 mg of the powder were weighed and added to 1.0 mL of a 70% aqueous methanol solution. This mixture was stored at 4 °C overnight and centrifuged at 13,800× *g* for 10 min. Ultimately, the supernatant was finally filtered through a 0.22 µm micropore filter. In this study, an Agilent G6400 triple quadrupole mass spectrometer coupled with an Agilent 1290II UPLC (Agilent Technologies, Santa Clara, CA, USA) system was employed for multiple reaction monitoring (MRM). The operating parameters and gradient program were consistent with those established in our previous study [30]. The specific conditions were as follows: column temperature: 35 °C; column: C18 (1.8 µm, 100 mm × 2.1 mm); mobile phase: elution A (aqueous solution of 0.5% acetic acid and 5 mM ammonium acetate), elution B (100% acetonitrile); sample injection volume: 3 μL; flow rate: 0.3 mL/min. The relative content of each metabolite in the samples was expressed as the chromatographic peak area.

### 2.8. Statistical Analysis

The protein–protein interaction network for CqDofs was predicted based on the STRING website (https://cn.string-db.org/, accessed on 12 November 2024), which contains known and predicted protein–protein interactions. All experiments were performed with 3 biological replicates, and TBtools Prism 8 software was used to draw graphs. The significance of the difference between each treatment group compared to the control was determined using one-way ANOVA in SPSS 26.0 (IBM Corp., Armonk, NY, USA); data were expressed as the means ± SD, and asterisks indicate significant differences between the treatments and the control: * *p* ≤ 0.05, ** *p* ≤ 0.01, *** *p* ≤ 0.001.

## 3. Results

### 3.1. Identification, Physicochemical Properties, and Chromosomal Locations Analysis of CqDof Genes in Quinoa

A total of 36 *CqDof* genes were identified after removing duplicated sequences and incomplete structural domains and named *CqDof1*–*CqDof36* according to their gene ID (Table 1). The physicochemical properties of *CqDofs* revealed that the amino acid sequence lengths ranged from 110 (*CqDof29*) to 558 (*CqDof5*), and the molecular weight (MW) varied from 12.22 kDa (*CqDof29*) to 59.1 kDa (*CqDof5*), exhibiting a considerable degree of variability. The theoretical isoelectric point (PI) value of the *CqDofs* ranged from 4.52 (*CqDof14*) to 11.46 (*CqDof16*); *CqDof8*, *CqDof12*, *CqDof13*, *CqDof14*, *CqDof15*, *CqDof18*, *CqDof22*, *CqDof26*, *CqDof30*, *CqDof31*, and *CqDof35* were classified as acidic proteins due to their isoelectric point value being less than 7, while the others were basic proteins. The instability index of *CqDofs* ranged from 32.55 (*CqDof20*) to 85.15 (*CqDof16*), and all proteins were unstable except for CqDof20. Furthermore, the grand average hydropathicity (GRAVY) value for all CqDof proteins was negative, indicating they were hydrophilic, and subcellular localization prediction showed that all CqDof proteins were nuclear-localized.

**Figure 1:**
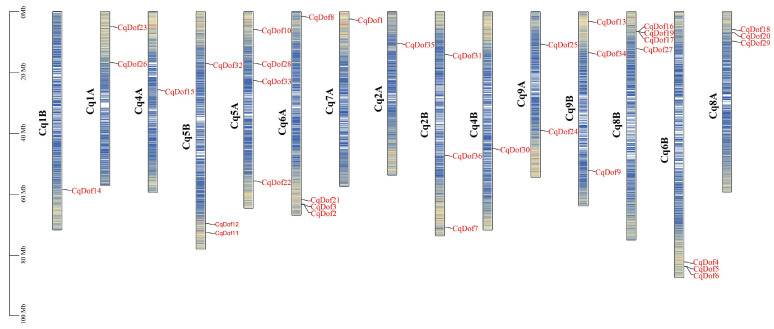
Chromosome localization of *CqDof* genes in quinoa. The scale on the left denotes chromosome length. Suffixes A and B for the chromosome number indicate subgenomes A and B in the quinoa genome, respectively.

The chromosomal locations of the 36 *CqDofs* were determined based on the quinoa genome annotation file. The results showed that 36 *CqDof* genes were unevenly distributed on 15 chromosomes, but no *CqDofs* were present on Cq3A, Cq3B, and Cq7B (Figure 1). The *CqDofs* were predominantly distributed at both ends of the chromosome, with a sparser presence in the middle regions. The highest numbers of genes (four) were distributed on Cq5A, Cq6A, and Cq8B, followed by three genes on Cq5B, Cq2B, Cq9B, Cq6B, and Cq8A and one gene on Cq1B, Cq4A, Cq7A, Cq2A, and Cq4B. In addition, some genes were physically adjacent to each other, such as *CqDof16*, *CqDof17*, and *CqDof19* (Figure 1), suggesting they may have similar functions.

### 3.2. Phylogenetic Relationship and Classification of CqDof Genes in Quinoa

**Figure 2:**
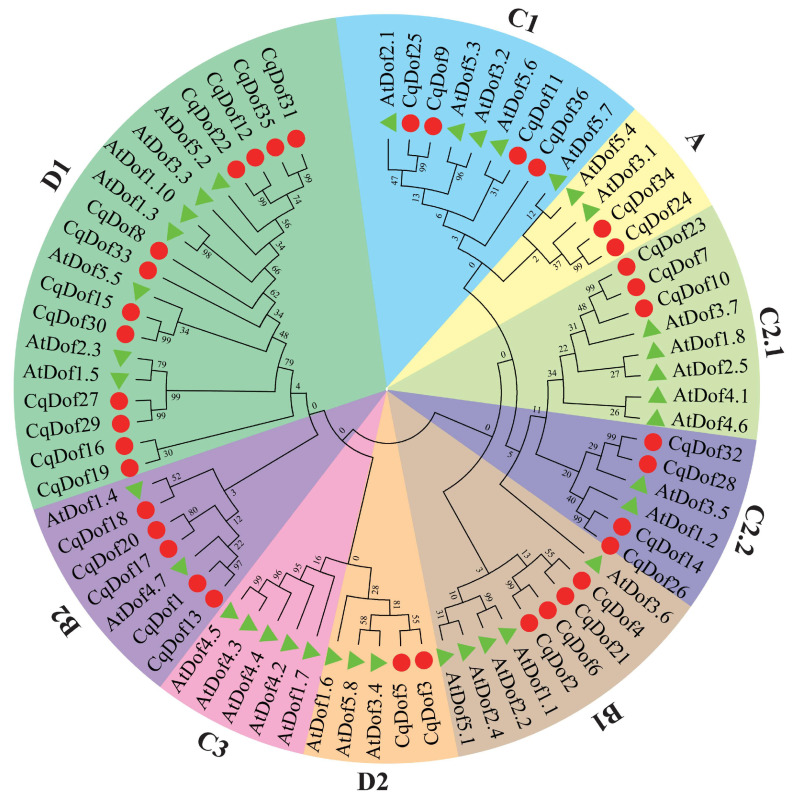
Phylogenetic relationships of Dof proteins from quinoa and *A. thaliana.* The *Dof* genes of quinoa and *A. thaliana* are represented by circles and triangles, respectively. The phylogenetic tree was constructed using the neighbor-joining method. The best evolutionary model JTT + G + I + F calculated through MEGA X was selected with the bootstrap value of 1000. Numbers in the tree represent the bootstrap values of the nodes and branches.

To elucidate the evolutionary relationship among *CqDofs* in quinoa, an NJ phylogenetic tree was constructed using the complete amino acid sequences of CqDofs and AtDofs using MEGA software (Figure 2). The results revealed that CqDof proteins were categorized into 9 subgroups (A, B1, B2, C1, C2.1, C2.2, C3, D1, D2), and the distribution of individuals within each subgroup varied greatly. The subgroup D1 contained the highest number of CqDof proteins (12 members), followed by B2 (5 members), B1, C1, and C2.2 (4 members), C2.2 (3 members), and A and D2 (2 members). Significantly, no CqDof proteins from the C3 subfamily were found to co-occur with AtDof proteins on the same branch, indicating that some *CqDof* genes have undergone genetic evolutionary changes and may be lost in quinoa. Furthermore, some gene pairs, including *CqDof18* with *AtDof1.4*, *CqDof11* with *AtDof5.6*, were directly homologous between *A. thaliana* and quinoa, suggesting potential similarities in their evolutionary patterns and gene functions.

### 3.3. Gene Duplication and Syntenic Analysis of CqDof Genes in Quinoa

**Figure 3:**
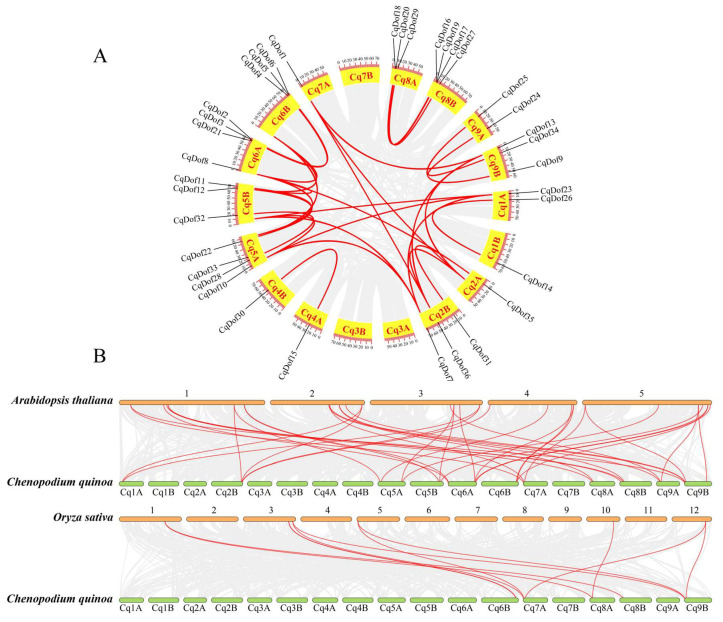
Gene duplication and syntenic analysis of the *CqDof* genes in quinoa. (**A**) Gene duplication analysis of the *CqDof* genes. The grey lines represent all collinear blocks in the quinoa genome, and the red lines represent the segmental replication line relationship between the *CqDof* genes. The number on the perimeter of the circle represents the length of the chromosome. (**B**) Syntenic analysis of the *Dof* genes among *C. quinoa*, *A. thaliana*, and *O. sativa*. The gray lines in the background show collinearity between the *C. quinoa* and *A. thaliana*, *O. sativa* genomes. The red lines denote syntenic *Dof* gene pairs between quinoa and other plant genomes.

Gene duplication has an important role in the evolution of a gene family and the exploration of gene function. Therefore, this research analyzed the tandem and fragment duplication events to explore the evolutionary relationship among 36 *CqDof* genes. The results showed that 25 pairs of segmental gene replication were detected, with no tandem duplication events observed among the *CqDof* genes, suggesting that segmental duplication might be a major driving force in the evolution of the *CsDof* gene family (Figure 3A). In addition, the evolutionary trend and genetic relationship of the *Dof* family in quinoa, *A. thaliana*, and *O. sativa* were also analyzed, and it was found that quinoa had a strong synteny relationship with *A. thaliana* and a simpler synteny relationship with rice (Figure 3B). Among them, chromosome Cq6B exhibited the highest number of homologous gene pairs with *A. thaliana*, while chromosomes Cq7A and Cq9B had the most homologous gene pairs with *O. sativa*. Collectively, these findings provide foundational insights into the evolution and origin of species.

### 3.4. Gene Structure, Conserved Domain, and Motif Analysis of the CqDof Genes

**Figure 4:**
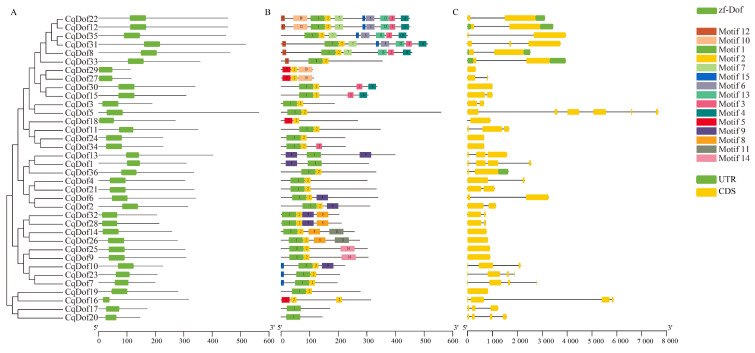
Analysis of conserved domains (**A**), motif (**B**), and gene structure (**C**) of the *CqDof* genes. Different color modules represent different elements. UTR: untranslated region, CDS: coding sequence, lines indicate introns.

Consistent with expectations, the Batch CD-Search analysis revealed that all 36 CqDof proteins possess a highly conserved Dof domain (Figure 4A), confirming the reliability of the gene identification outcomes. In this study, we utilized MEME to identify 15 conserved motifs within the CqDof proteins, and each *CqDof* contained 1–10 conserved motifs, with *CqDof12* and *CqDof22* exhibiting the highest number of motifs. Furthermore, the majority of *CqDofs* contained both motif 1 and motif 2, and the *CqDof* genes located on the same phylogenetic branch exhibited similar motif compositions, highlighting the structural homology among the CqDof proteins (Figure 4B).

Similarly, we mapped the distribution of CDS, UTR, and introns of *CqDof* genes to enhance the understanding of their structural features. The *CqDof* genes exhibited a variable intron count, with *CqDof5* featuring the longest intron. Notably, *CqDof29*, *CqDof30*, *CqDof24*, *CqDof34*, *CqDof9*, *CqDof25*, *CqDof26*, *CqDof14*, and *CqDof19* lacked introns and UTRs, consisting exclusively of exons. The *CqDof* genes belonging to the same subfamily exhibited comparable exon and intron counts. For instance, *CqDof7* and *CqDof23* each contain four exons and three introns (Figure 4C), indicating that members of the same subfamily are highly conserved in gene structure, providing a foundation for future functional studies of this gene family.

### 3.5. Promoter cis-Acting Element Analysis of the CqDof Genes

**Figure 5:**
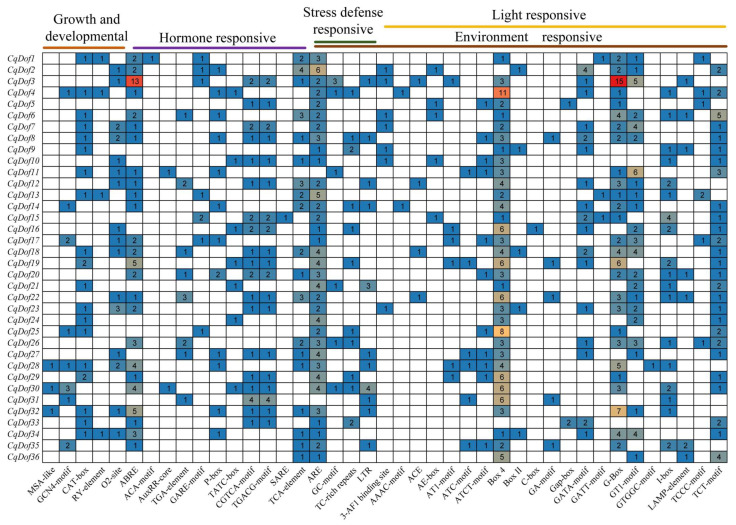
Distribution of cis-acting elements in the promoter regions of the *CqDof* genes. The color intensity and the numbers in the grids indicate the numbers of cis-acting elements.

Transcription factors interact with cis-acting elements in promoter regions to initiate gene transcription, potentially modulating the expression of specific genes. To further understand the potential regulatory mechanisms of *CqDofs* in response to abiotic stress, hormone treatment, and plant growth and development, we analyzed the specific cis-elements of *CqDofs*. The results showed that *CqDof* gene cis-acting elements can be primarily categorized into three types: plant growth and developmental, hormone-responsive, and environmentally responsive (Figure 5). The plant growth and developmental types included cell cycle regulation, circadian control, endosperm expression, meristem expression, seed-specific regulation, and the Box and O2-site elements were most distributed in the *CqDof* genes. The hormone-responsive types included elements involved in salicylic acid, methyl jasmonate (MeJA), gibberellin, auxin, and abscisic acid. Among these, the ABRE element was most abundant in *CqDof* gene promoters. The environmentally responsive types included elements associated with light responsiveness, stress defense responsiveness, low-temperature responsiveness, and anaerobic induction responsiveness, with the Box 4 element being the most abundant, followed by the G-Box and TCT-motif. In addition, a total of 804 cis-acting elements were identified in *CqDofs*, including 61 growth and developmental elements, 193 hormone-responsive elements, and 550 environmentally responsive elements (Figure 5). Notably, all *CqDof* gene promoter regions contained light-responsive elements, and these were the most numerous elements of the environmentally responsive type, suggesting that the *CqDof* genes likely play a role in regulating plant growth, metabolism, hormone signaling, or other physiological processes by participating in the plant response to light signals.

### 3.6. Expression Pattern of the CqDof Genes in Various Tissues Under Abiotic Stresses

**Figure 6:**
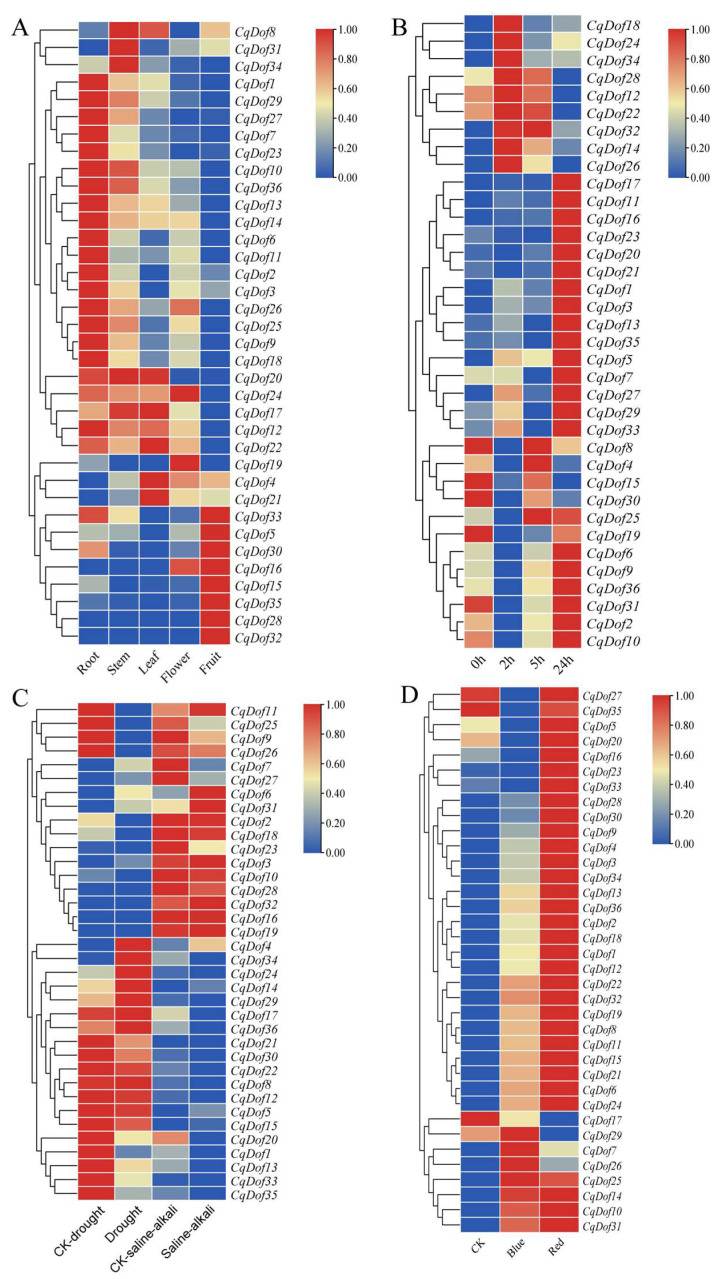
Heatmap display of the *CqDof* genes in quinoa. (**A**) Expression profile of *CqDofs* in roots, stems, leaves, flowers, and fruits. (**B**) Expression profile of *CqDofs* under salt stress. (**C**) Expression profile of *CqDofs* under drought and saline-alkali stress. (**D**) Expression profile of *CqDofs* under red and blue light treatments. The color gradient represents log2 fold change, ranging from higher (red) to lower (blue). CK: normal condition.

To assess the expression pattern of *CqDofs* in different tissues of quinoa, we constructed expression profiles for 36 *CqDofs* in roots, stems, leaves, flowers, and fruits using RNA-seq data (FPKM values converted to log2FC). The results revealed tissue-specific expression profiles for *CqDofs*, with the majority of *CqDofs* showing high expression in roots (Figure 6A). Conversely, *CqDof16*, *CqDof28*, and *CqDof32* were not expressed in roots, stems, or leaves, indicating their potential lack of involvement in quinoa’s growth and development. In addition, *CqDof12*, *CqDo17*, *CqDo22*, *CqDo24*, and *CqDof34* exhibited high expression in stems and leaves, while *CqDof28*, *CqDo35*, *CqDo5*, *CqDo15*, *CqDo32*, *CqDof30*, and *CqDof33* were markedly expressed in fruits, with low *CqDof* expression observed in flowers (Figure 6A).

The expression profiles of *CqDofs* under several abiotic stresses and different light treatments were also examined in this study. The findings indicated a dynamic expression pattern of *CqDofs* during salt stress, with most genes, including *CqDof17*, *CqDof11*, *CqDof16*, and *CqDof23*, being upregulated in response (Figure 6B). Some *CqDof* genes also responded to drought stress, with *CqDof11, CqDof25, CqDof9, CqDof26, CqDof20, CqDof1, CqDof13, CqDof33*, and *CqDof35* were downregulated, while *CqDof4, CqDof34, CqDof24, CqDof14*, and *CqDof29* were upregulated (Figure 6C). Saline-alkali stress resulted in an increased expression of *CqDof6, CqDof31*, and *CqDof11* and a decreased expression of *CqDof25, CqDof9, CqDof7, CqDof27*, and *CqDof23* (Figure 6C). Given that all *CqDof* genes contain light-responsive elements, this implies that these genes may play a significant regulatory role in light response. Consequently, this study conducted an RNA-Seq analysis on quinoa samples treated with red and blue light. The results revealed that the majority of genes exhibited an enhanced expression under both blue and red light, with a more pronounced increase observed following red light treatment (Figure 6D). Notably, some genes, such as *CqDof6*, *CqDof7*, and *CqDof20*, were responsive to these treatments, suggesting a potential role for them in adapting to salt, drought, saline-alkali stress, and light treatment.

### 3.7. Expression Analysis of the CqDof Genes Under Abiotic Stress and Light Treatment

**Figure 7:**
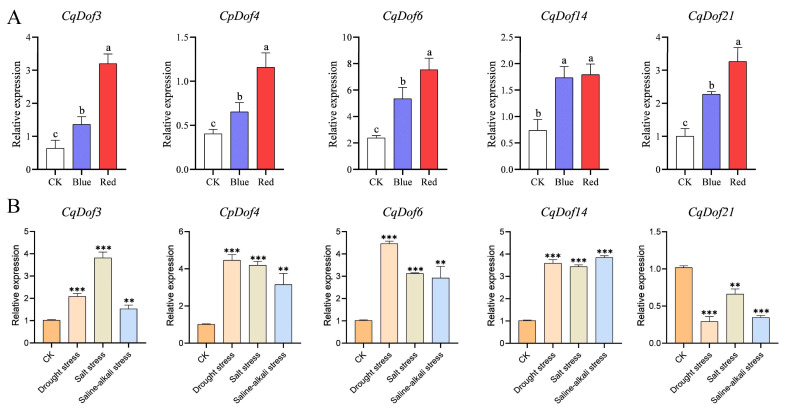
The relative expression levels of the *CqDof* genes in leaves under light treatment (**A**) and under drought, salt, and saline-alkali stress (**B**). The error bars represent standard deviations (*n* = 3). Note: Different lowercase letters represent significant differences (*p* < 0.05) followed by Tukey’s multiple range test. **, and *** represent *p* < 0.01, *p* < 0.001 using Student′s *t*-test. CK: normal growth condition; Blue: blue light treatment; Red: red light treatment.

DEGs in response to light treatment were identified based on the criteria of log2 fold change ≥ 1 and *p*-value < 0.05, leading to the identification of five genes (*CqDof3*, *CqDof4*, *CqDof6*, *CqDof14*, *CqDof21*) that were all upregulated by the light treatment. Subsequently, the expression levels of five DEGs were verified using RT-PCR, which confirmed the RNA-seq data, indicating that the data were accurate and reliable (Figure 7A). To further explore the functions of the above 5 *CqDof* genes under abiotic stress, RT-PCR was used to analyze their expression profiles under abiotic stress in this study. The results indicated that these *CqDof* genes exhibited distinct responses to drought, salt, and saline-alkali stresses (Figure 7B). Specifically, *CqDof3*, *CqDof4*, *CqDof6*, and *CqDof14* exhibited the most significant upregulation in expression levels under salt stress, drought stress, and saline-alkali stress, respectively, with 3.75-fold, 4.38-fold, 4.39-fold, and 3.78-fold increases. Notably, *CqDof14* showed a consistent response pattern under three different abiotic stresses, suggesting its potential role as a common factor in plant adaptation to multiple stress conditions. Furthermore, the expression of *CqDof21* was significantly downregulated under drought, salt, and saline-alkali stresses, with 3.49-fold, 1.54-fold, and 2.94-fold reductions, respectively. These results suggest that *CqDof21* may play an inhibitory role in the regulation of plant responses to abiotic stress.

### 3.8. Analysis of the Flavonoid Content After Light Treatment

**Figure 8:**
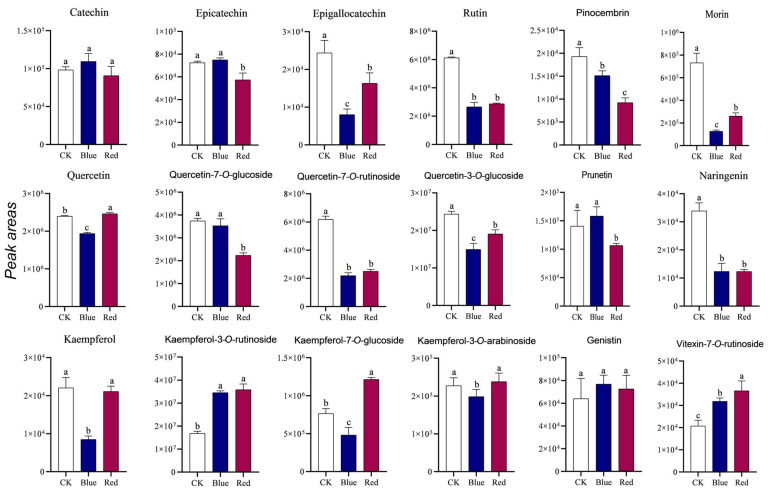
Statistical analysis of the relative contents of flavonoids under red and blue light treatments. The error bars represent standard deviations (*n* = 3). Different lowercase letters represent significant differences (*p* < 0.05) followed by Tukey’s multiple range test. CK: normal growth condition; Blue: blue light treatment; Red: red light treatment.

Quinoa is rich in a variety of flavonoid compounds, including rutin, catechin, epicatechin, morin, quercetin, and kaempferol [31]. Previous studies demonstrated that light treatment can influence the accumulation of flavonoids in plants [32]. Consequently, this study employed LC–MS to assess the levels of certain flavonoids in quinoa subjected to red and blue light treatments. The results revealed that the contents of most flavonoids decreased under blue and red light treatments. Specifically, blue light treatment led to a significant reduction in the levels of epigallocatechin, rutin, naringenin, morin, pinocembrin, quercetin-7-O-rutinoside, quercetin-3-O-glucoside, and naringenin by factors of 2.78, 2.31, 2.74, 5.80, 1.28, 2.82, 1.63, and 2.74, respectively. In contrast, red light treatment resulted in decreases in their concentrations by factors of 1.48, 2.13, 2.76, 2.82, 2.09, 2.47, 1.28, and 2.76, respectively (Figure 8). Additionally, the concentrations of kaempferol-3-O-rutinoside and vitexin-7-O-rutinoside were found to be approximately 2.01-fold and 1.54-fold greater under blue light conditions, and 2.13-fold and 1.76-fold greater under red light conditions, respectively. It is noteworthy that epicatechin, quercetin-7-O-glucoside, and prunitrin showed no significant alteration under blue light treatment, while catechin and genistin exhibited no significant change under both red and blue light treatments (Figure 8).

### 3.9. Correlation and Interaction Analysis Between CqDofs and Flavonoids

**Figure 9:**
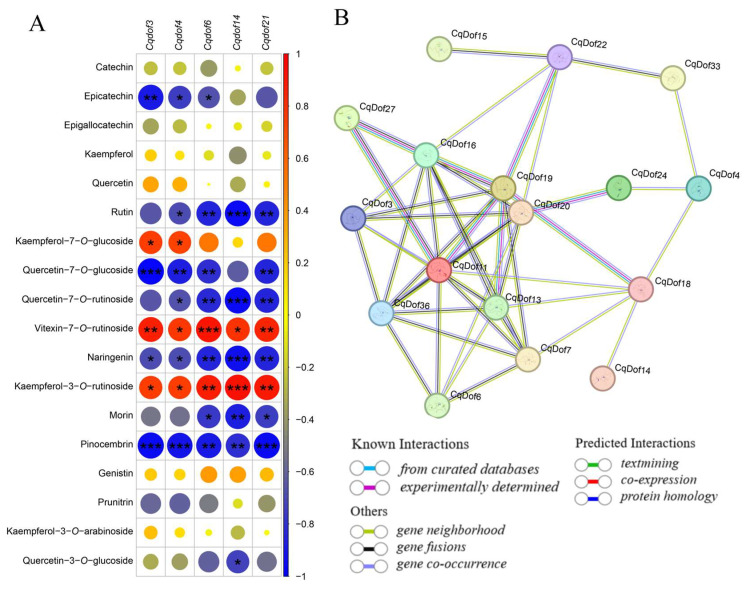
Pearson correlation and protein interaction network analysis. (**A**) Pearson correlation analysis between *CqDofs* and flavonoids. The color intensity (ranging from red to blue) and the circle diameter represent the correlation strength depicted as the r-value. Circles marked with asterisks denote the statistical significance of the correlations, where *, **, and *** represent *p* < 0.05, *p* < 0.01, *p* < 0.001, respectively. (**B**) Interaction network analysis of the CqDof proteins. The numbers (*CqDof* gene number) in brackets represent the corresponding orthologs in quinoa, where distinct line colors signify the categories of interactions as defined in the legend.

The correlation analysis revealed that *CqDof3*, *CqDof4*, *CqDof6*, *CqDof14*, and *CqDof21* were significantly negatively correlated with rutin, pinocembrin, naringenin, quercetin-7-O-rutinoside, and quercetin-7-O-glucoside, and significantly positively correlated with vitexin-7-O-rutinoside and kaempferol-3-O-rutinoside. However, there was no significant correlation between these 5 *CqDof* genes and catechin, epigallocatechin, kaempferol, quercetin, genistin, prunitrin, and kaempferol-3-O-arabinoside (Figure 9A). These results indicate that blue light and red light potentially regulate flavonoid biosynthesis in quinoa by modulating the *CqDof* genes’ expression levels. Meanwhile, the Dof domain in Dof TFs is a key domain that can mediate protein–protein interactions, implying that Dof TFs may also play a role by forming protein complexes. To explore potential interactions between *CqDofs*, an interaction network was constructed using the STRING database, which was predicated on the orthologous relationships between CqDofs and AtDofs. The results indicated that there were protein interactions between homologous CqDofs corresponding to AtDofs, such as DOF3.5 (CqDof14/–26/–28/–32) interacting with DOF1.4 (CqDof18) and DOF3.4 (CqDof3) interacting with DOF3.6 (CqDof16)/DOF3.7 (CqDof19)/DOF5.1 (CqDof20)/DOF5.6 (CqDof11)/DOF2.4 (CqDof3.6). Furthermore, each of the five identified CqDofs (CqDof3, CqDof4, CqDof6, CqDof14, CqDof21) were found to interact with additional members of the quinoa Dof family (Figure 9B). In brief, the interaction network of *CqDofs* exhibits a potential intricacy in their associations, indicating a potential role for the *CqDof* genes in modulating flavonoid biosynthesis in quinoa through interactions with other CqDof proteins.

## 4. Discussion

Dof proteins are plant-specific transcription factors. In plants, Dof transcription factors are not only associated with vascular development, seed germination, pollen development, hormone response, and secondary metabolite synthesis, but also play important roles in abiotic stress tolerance, including resistance to salt, drought, high temperature, and cold [33]. Quinoa, as a nutritionally prominent functional health food, is rich in polyphenols, flavonoids, saponins, and polysaccharides, as well as essential nutrients such as vitamins, essential amino acids, and minerals (K, P, Mg, Ca, Zn, Fe), which possess antioxidant, hypoglycemic, lipid-lowering, anti-inflammatory, immune system-related, and cardiovascular disease-preventive physiological activities [25]. The *Dof* gene family has been extensively studied in many plant species, but no studies have specifically addressed the *Dof* gene family in quinoa. In this study, 36 *CqDof* genes were identified in the quinoa genome, a number comparable to the *Dof* genes reported in *A. thaliana* (36), *Solanum tuberosum* (35), *Solanum lycopersicum* (34), and *Piper nigrum* (33). However, the number of genes was lower than that in *Brassica napus* (117), *Triticum aestivum* (108), and *Saccharum officinarum* (119), and the number of transcription factors varied considerably between monocotyledonous and dicotyledonous plants, which may be related to the fact that they have experienced different evolutionary pressures in the expansion and contraction of their gene families [6]. Additionally, most CqDof proteins are basic, hydrophilic, and unstable, which is similar to the physicochemical properties of CqDof proteins in *Medicago polymorpha* [34] and *Camelina sativa* [35], suggesting a high degree of conservation of *Dof* genes across different species.

The chromosomal distribution of the 36 *CqDof* genes in quinoa was uneven, and the number of genes did not correlate with chromosome size. Notably, no *CqDof* genes were found on chromosomes Cq3A, Cq3B, and Cq7B, which may be related to fragment loss or chromosomal translocation events during the evolutionary process. Phylogenetic analysis showed that 36 *CqDof* genes were divided into 9 subgroups based on the Dof proteins from quinoa and *Arabidopsis*, consistent with the results for *Brassica napus* [36] and watermelon [37]. Multiple homologs were identified between the *CqDof* and *AtDof* genes across various subfamilies, including *CqDof18* with *AtDof1.4* (B2 subfamily) and *CqDof11* with *AtDof5.6* (C1 subfamily). *AtDof5.6* (AT5G62940) exhibited high expression levels in *Arabidopsis* stems and leaves and modulated the stem size through the regulation of vascular tissue development, suggesting a potential analogous biological function for *CqDof11* [38]. Interestingly, the C3 subfamily has only *AtDofs* but no *CqDofs*, which also occurs in *Vaccinium corymbosum*, indicating that the *CqDof* gene family may have undergone a contraction event during evolution [39]. Gene structure analysis revealed that all CqDof proteins contained a Dof domain, and *CqDof* genes from different subfamilies differed significantly in motif composition, while those from the same subgroup had a similar motif composition. Furthermore, the majority of *CqDofs* contained only one intron, and 7 *CqDofs* were intronless, which was similar to the findings in sweet potatoes [6]. Genes without introns have been reported to be more likely to function in abiotic stress responses such as drought and salinity, but the specific functions of intron deficiency in plant resistance to abiotic stresses need to be further studied [40].

Gene duplication is one of the key drivers of plant genome evolution, plays an essential role in the expansion of gene family members, and promotes specificity and diversity of gene functions [41]. In this study, 25 pairs of *CqDof* genes were involved in fragment replication, but no tandem duplication events were detected, indicating that fragment repetition events played a leading role in *CqDof* gene amplification, consistent with the results observed in roses [42] and Tartary buckwheat [43]. Furthermore, syntenic analysis revealed a significant number of orthologous gene pairs between quinoa and *Arabidopsis*, implying a closer evolutionary relationship between these two species.

Gene expression analysis in different tissues and various stress conditions is a critical approach for elucidating gene function [44]. In this study, the *CqDof* genes exhibited differential tissue-specific expression, with a majority being highly expressed in roots, which was consistent with the expression profiles observed in pepper and cucumber [45,46]. Transcriptomic analysis demonstrated that salt, drought, and saline-alkali stresses differentially induced *CqDof* gene expression, revealing that these genes may serve distinct functions in the response to diverse abiotic stresses. For example, *CqDof14* was induced by all three different abiotic stresses, suggesting that it plays an important role in improving quinoa stress tolerance. These findings are important for understanding the response mechanism of *Dof* genes to abiotic stress in crops and their role in agronomic traits. Furthermore, cis-elements, serving as specific binding sites for transcription factors, are essential for the regulation of gene expression [41]. Therefore, this investigation explored the type and number of cis-elements in the promoter of *CqDof* genes. The results showed numerous developmental, hormonal, and stress-responsive cis-elements, including ABRE, ARE, LTR, CAT-box, P-box, Box-4, G-box, GT1-motif, and TCT-motif. Notably, the promoter regions exhibited abundant light-responsive elements. Previous studies demonstrated that Dof TFs influenced plant growth, development, and the biosynthesis of secondary metabolites by responding to light signaling. For instance, Gao et al. reported that *Arabidopsis* Dof TFs, specifically CDFs, modulated light responses by promoting hypocotyl cell elongation [47]. Additionally, DAG2 was identified as a positive regulator in light-induced seed germination, with red light markedly suppressing germination rates in DAG2 mutant seeds [48]. Huang et al. discovered that red light enhanced the expression of Dof TFs and stimulated carotenoid synthesis in *Citrus paradisi* [49]. However, the response of Dof TFs and alterations in secondary metabolites in quinoa under different light stresses have not been studied. In this study, transcriptome data analysis indicated that red light and blue light induced the expression of most *CqDof* genes, with red light eliciting a stronger response. Further investigation identified 5 *CqDof* genes (*CqDof3*, *CqDof4*, *CqDof6*, *CqDof14*, and *CqDof21*) that exhibited significant expression changes following both blue and red light treatments, as confirmed by RT-PCR assays, suggesting their critical regulatory roles in light response.

Numerous studies have demonstrated that red and blue light can induce flavonoid synthesis. Specifically, blue light induced flavonoid biosynthesis in *Epimedium sagittatum* [50], while red light enhanced the total flavonoid content in buckwheat sprouts [51]. In the present study, the content of various flavonoids, except for catechin and genistin, was altered by monochromatic blue and red light treatments, including changes in rutin, naringenin, morin, pinocembrin, and quercetin-7-O-rutinoside. Both blue and red light treatments influenced secondary metabolite accumulation in quinoa, but their effects differed significantly. For example, red light significantly increased the content of kaempferol-3-O-glucoside, whereas blue light negatively affected the accumulation of this compound. Red light increased the content of vitexin-7-O-rutinoside more effectively than blue light, which is also consistent with previous studies [52]. Correlation analysis between the 5 *CqDof* genes and 18 flavonoids indicated significant relationships, such as a positive correlation between *CqDof14* and kaempferol-3-O-rutinoside, and a negative correlation between *CqDof6* and rutin. These findings suggest varying functional roles among the *CqDof* genes in the regulation of flavonoid synthesis, as well as potential differences in the regulatory capacity. Additionally, protein interaction network predictions showed that the CqDof proteins can interact with one another, exemplified by the potential interaction between DOF3.5 (CqDof14/-26/-28/-32) and DOF1.4 (CqDof18). This highlights the complex interaction network among the CqDof proteins, but the precise mechanisms of these interactions require further investigation. Overall, these results provide a theoretical basis for the elucidation of the specific mechanisms by which the *CqDof* genes regulate flavonoid synthesis under various light conditions, which will be crucial for future efforts to enhance flavonoid production in quinoa, as well as for crop quality enhancement.

## 5. Conclusions

In summary, this study identified the *CqDof* gene family in quinoa at the genome-wide level, and further investigated their physicochemical properties, phylogenetic relationships, gene structures, and cis-acting elements. In addition, blue and red light treatments significantly altered *CqDof* gene expression and flavonoid accumulation, while *CqDof3*, *CqDof4*, *CqDof6*, *CqDof14*, and *CqDof21* were also affected by abiotic stresses. Therefore, the results of this study will help to study the function of the *CqDof* gene family in quinoa and the mechanism of regulating flavonoid synthesis and provide a theoretical basis for screening genetic improvement genes to enhance quinoa stress tolerance.

## Figures and Tables

**Table 1:** The basic information of *CqDof* genes in quinoa.

Gene ID	Gene Name	Length (Aa)	MV (kDa)	PI	Instability Index	GRAVY	Aliphatic Index	Subcellular Location
AUR62001807	*CqDof1*	305	32.51	9.06	44.57	−0.742	49.61	Nucleus
AUR62001970	*CqDof2*	196	21.93	9.38	43.76	−0.886	52.76	Nucleus
AUR62001976	*CqDof3*	186	19.17	8.72	50.76	−0.356	55.97	Nucleus
AUR62003691	*CqDof4*	291	31.13	9.40	51.63	−0.604	61.07	Nucleus
AUR62003828	*CqDof5*	558	59.10	9.09	56.97	−0.253	66.49	Nucleus
AUR62003833	*CqDof6*	337	35.84	9.18	49.13	−0.620	53.23	Nucleus
AUR62004520	*CqDof7*	196	21.93	9.38	43.76	−0.886	52.76	Nucleus
AUR62005809	*CqDof8*	457	50.13	5.60	50.34	−0.765	55.30	Nucleus
AUR62006501	*CqDof9*	304	33.90	8.07	52.68	−0.838	55.49	Nucleus
AUR62007029	*CqDof10*	223	25.32	9.44	51.32	−0.904	55.96	Nucleus
AUR62008205	*CqDof11*	346	36.87	8.03	50.31	−0.640	48.50	Nucleus
AUR62008425	*CqDof12*	450	49.65	5.13	55.16	−0.901	48.56	Nucleus
AUR62009593	*CqDof13*	398	42.41	6.65	51.28	−0.816	49.30	Nucleus
AUR62013510	*CqDof14*	255	28.01	4.52	57.45	−0.656	51.29	Nucleus
AUR62014301	*CqDof15*	304	33.44	6.03	45.24	−0.560	62.96	Nucleus
AUR62014841	*CqDof16*	313	34.96	11.46	85.15	−0.708	67.48	Nucleus
AUR62014843	*CqDof17*	169	18.78	8.31	46.39	−0.805	47.46	Nucleus
AUR62016967	*CqDof18*	267	29.85	6.37	57.96	−0.910	47.12	Nucleus
AUR62017038	*CqDof19*	276	30.08	8.22	56.50	−0.691	62.10	Nucleus
AUR62017040	*CqDof20*	143	16.08	8.24	32.55	−0.859	42.45	Nucleus
AUR62018002	*CqDof21*	332	36.19	8.07	45.31	−0.772	55.84	Nucleus
AUR62021670	*CqDof22*	449	49.55	5.13	54.30	−0.902	48.66	Nucleus
AUR62022735	*CqDof23*	204	22.64	9.60	48.23	−0.948	49.75	Nucleus
AUR62023916	*CqDof24*	223	23.49	9.32	56.20	−0.535	59.15	Nucleus
AUR62025032	*CqDof25*	301	33.49	8.07	54.93	−0.829	56.05	Nucleus
AUR62026763	*CqDof26*	274	30.31	4.61	58.22	−0.626	51.64	Nucleus
AUR62027677	*CqDof27*	113	12.69	9.26	50.58	−0.536	56.99	Nucleus
AUR62030727	*CqDof28*	210	23.52	9.11	47.08	−0.592	60.76	Nucleus
AUR62031206	*CqDof29*	110	12.22	8.69	45.05	−0.592	45.27	Nucleus
AUR62034094	*CqDof30*	335	37.29	6.55	51.77	−0.626	65.25	Nucleus
AUR62034427	*CqDof31*	512	56.46	5.47	45.21	−0.684	64.20	Nucleus
AUR62036367	*CqDof32*	202	22.65	8.96	47.13	−0.760	51.14	Nucleus
AUR62036527	*CqDof33*	353	38.87	7.14	61.18	−0.620	61.81	Nucleus
AUR62038328	*CqDof34*	224	23.79	9.41	53.53	−0.631	51.47	Nucleus
AUR62039802	*CqDof35*	442	48.70	6.80	43.72	−0.778	53.46	Nucleus
AUR62041860	*CqDof36*	331	35.74	8.93	53.75	−0.662	57.73	Nucleus

MW: molecular weight; PI: isoelectric point; GRAVY: grand average hydropathicity.

## Data Availability

The quinoa genome data used in this study can be found at https://datadryad.org/stash/dataset/doi:10.5061/dryad.kwh70rz70, accessed on 15 September 2024. The transcriptome data were deposited in the NCBI database under accession numbers PRJNA658178, PRJNA578698, and PRJNA636120.

## Supplementary Materials

The following supporting information can be downloaded at: https://www.mdpi.com/article/10.3390/biology14040446/s1, Table S1: RT-PCR primers for the *CqDof* genes.

## References

[1] Qian G., Li X., Zhang H., Zhang H., Zhou J., Ma X., Sun W., Yang W., He R., Wahab A.T. (2023). Metabolomics analysis reveals the accumulation patterns of flavonoids and phenolic acids in quinoa (*Chenopodium quinoa* Willd.) grains of different colors. Food Chem. X.

[2] Sampaio S.L., Fernandes A., Pereira C., Calhelha R.C., Sokovic M., Santos-Buelga C., Barros L., Ferreira I. (2020). Nutritional value, physicochemical characterization and bioactive properties of the Brazilian quinoa brs piabiru. Food Funct..

[3] Melini V., Melini F. (2021). Functional components and anti-nutritional factors in gluten-free grains: A focus on quinoa seeds. Foods.

[4] Pereira E., Cadavez V., Barros L., Encina-Zelada C., Stojkovic D., Sokovic M., Calhelha R.C., Gonzales-Barron U., Ferreira I. (2020). *Chenopodium quinoa* Willd. (quinoa) grains: A good source of phenolic compounds. Food Res. Int..

[5] Zhang J., Liu B., Li J., Zhang L., Wang Y., Zheng H., Lu M., Chen J. (2015). Hsf and Hsp gene families in populus: Genome-wide identification, organization and correlated expression during development and in stress responses. BMC Genom..

[6] Zhang C., Dong T., Yu J., Hong H., Liu S., Guo F., Ma H., Zhang J., Zhu M., Meng X. (2023). Genome-wide survey and expression analysis of Dof transcription factor family in sweetpotato shed light on their promising functions in stress tolerance. Front. Plant Sci..

[7] Sellami M.H., Pulvento C., Lavini A. (2020). Agronomic practices and performances of quinoa under field conditions: A systematic review. Plants.

[8] Zhu X., Wang B., Liu W., Wei X., Wang X., Du X., Liu H. (2023). Genome-wide analysis of AP2/ERF gene and functional analysis of CqERF24 gene in drought stress in quinoa. Int. J. Biol. Macromol.

[9] Sun W., Wei J., Wu G., Xu H., Chen Y., Yao M., Zhan J., Yan J., Wu N., Chen H. (2022). CqZF-HD14 enhances drought tolerance in quinoa seedlings through interaction with CqHIPP34 and CqNAC79. Plant Sci..

[10] Tovar J.C., Berry J.C., Quillatupa C., Castillo S.E., Acosta-Gamboa L., Fahlgren N., Gehan M.A. (2022). Heat stress changes mineral nutrient concentrations in *Chenopodium quinoa* seed. Plant Direct.

[11] Tashi G., Zhan H., Xing G., Chang X., Zhang H., Nie X., Ji W. (2018). Genome-wide identification and expression analysis of heat shock transcription factor family in *Chenopodium quinoa* Willd. Agronomy.

[12] Hussin S.A., Ali S.H., Lotfy M.E., El-Samad E., Eid M.A., Abd-Elkader A.M., Eisa S.S. (2023). Morpho-physiological mechanisms of two different quinoa ecotypes to resist salt stress. BMC Plant Biol..

[13] Yang F., Fan Y., Wu X., Cheng Y., Liu Q., Feng L., Chen J., Wang Z., Wang X., Yong T. (2018). Auxin-to-gibberellin ratio as a signal for light intensity and quality in regulating soybean growth and matter partitioning. Front. Plant Sci..

[14] Zheng C., Ma J.Q., Ma C.L., Shen S.Y., Liu Y.F., Chen L. (2019). Regulation of growth and flavonoid formation of tea plants (*Camellia sinensis*) by blue and green light. J. Agric. Food. Chem..

[15] Manivannan A., Soundararajan P., Park Y.G., Jeong B.R. (2020). Physiological and proteomic insights into red and blue light-mediated enhancement of in vitro growth in *scrophularia kakudensis*—A potential medicinal plant. Front. Plant Sci..

[16] Huarancca Reyes T., Scartazza A., Castagna A., Cosio E.G., Ranieri A., Guglielminetti L. (2018). Physiological effects of short acute UVB treatments in *Chenopodium quinoa* Willd. Sci. Rep..

[17] Prado F.E., Perez M.L., González Y.J.A. (2016). Efectos de la radiación ultravioleta B (UV-B) sobre diferentes variedades de quinoa: II.- efectos sobre la síntesis de pigmentos fotosintéticos, protectores y azúcares solubles en condiciones controladas. Boletín La Soc. Argent. Botánica.

[18] Gupta S., Malviya N., Kushwaha H., Nasim J., Bisht N.C., Singh V.K., Yadav D. (2015). Insights into structural and functional diversity of Dof (DNA binding with one finger) transcription factor. Planta.

[19] Yanagisawa S., Schmidt R.J. (1999). Diversity and similarity among recognition sequences of Dof transcription factors. Plant J..

[20] Wei Z., Zhang H., Fang M., Lin S., Zhu M., Li Y., Jiang L., Cui T., Cui Y., Kui H. (2023). The Dof transcription factor COG1 acts as a key regulator of plant biomass by promoting photosynthesis and starch accumulation. Mol. Plant.

[21] Ramachandran V., Tobimatsu Y., Masaomi Y., Sano R., Umezawa T., Demura T., Ohtani M. (2020). Plant-specific dof transcription factors vascular-related dof1 and vascular-related dof2 regulate vascular cell differentiation and lignin biosynthesis in Arabidopsis. Plant Mol. Biol..

[22] Li Y., Tian M., Feng Z., Zhang J., Lu J., Fu X., Ma L., Wei H., Wang H. (2023). GhDof1.7, a dof transcription factor, plays positive regulatory role under salinity stress in upland cotton. Plants.

[23] Jarvis D.E., Ho Y.S., Lightfoot D.J., Schmockel S.M., Li B., Borm T.J., Ohyanagi H., Mineta K., Michell C.T., Saber N. (2017). The genome of *Chenopodium quinoa*. Nature.

[24] Rey E., Maughan P.J., Maumus F., Lewis D., Wilson L., Fuller J., Schmockel S.M., Jellen E.N., Tester M., Jarvis D.E. (2023). A chromosome-scale assembly of the quinoa genome provides insights into the structure and dynamics of its subgenomes. Commun. Biol..

[25] Qian G., Wang M., Zhou J., Wang X., Zhang Y., Liu Y., Zhu P., Han L., Li X., Liu C. (2024). Analysis of widely targeted metabolites of quinoa sprouts (*Chenopodium quinoa* Willd.) under saline-alkali stress provides new insights into nutritional value. Food Chem..

[26] Cai Z.Q., Gao Q. (2020). Comparative physiological and biochemical mechanisms of salt tolerance in five contrasting highland quinoa cultivars. BMC Plant Biol..

[27] Wang Y., Wu Y., Bao Q., Shi H., Zhang Y. (2024). Integrating physiology, transcriptome, and metabolome analyses reveals the drought response in two quinoa cultivars with contrasting drought tolerance. Int. J. Mol. Sci..

[28] Qian G., Meng X., Wang S., Mi Y., Qin Z., Liu T., Zhang Y., Wan H., Chen W., Sun W. (2023). Genome-wide identification of HSF gene family and their expression analysis in vegetative tissue of young seedlings of hemp under different light treatments. Ind. Crop Prod..

[29] Xiaolin Z., Baoqiang W., Xian W., Xiaohong W. (2022). Identification of the CIPK-CBL family gene and functional characterization of *CqCIPK14* gene under drought stress in quinoa. BMC Genom..

[30] Yang W., Qian G., Chen Y., Liu T., Wan H., Wang S., Meng X., Chen W., Su Y., Zhang Y. (2022). Profiling of polyphenols for in-depth understanding of *Tartary buckwheat* sprouts: Correlation between cultivars and active components, dynamic changes and the effects of ultraviolet B stress. Food Chem. X.

[31] Ren G., Teng C., Fan X., Guo S., Zhao G., Zhang L., Liang Z., Qin P. (2023). Nutrient composition, functional activity and industrial applications of quinoa (*Chenopodium quinoa* Willd.). Food Chem..

[32] Fu B., Ji X., Zhao M., He F., Wang X., Wang Y., Liu P., Niu L. (2016). The influence of light quality on the accumulation of flavonoids in tobacco (*Nicotiana tabacum* L.) leaves. J. Photochem. Photobiol. B.

[33] Zou X., Sun H. (2023). DOF transcription factors: Specific regulators of plant biological processes. Front. Plant Sci..

[34] Yang L., Min X., Wei Z., Liu N., Li J., Zhang Y., Yang Y. (2023). Genome-wide identification and expression analysis of the dof transcription factor in Annual Alfalfa *Medicago polymorpha*. Plants.

[35] Luo T., Song Y., Gao H., Wang M., Cui H., Ji C., Wang J., Yuan L., Li R. (2022). Genome-wide identification and functional analysis of Dof transcription factor family in *Camelina sativa*. BMC Genom..

[36] Lohani N., Babaei S., Singh M.B., Bhalla P.L. (2021). Genome-wide in silico identification and comparative analysis of dof gene family in *Brassica napus*. Plants.

[37] Zhou Y., Cheng Y., Wan C., Li J., Yang Y., Chen J. (2020). Genome-wide characterization and expression analysis of the Dof gene family related to abiotic stress in watermelon. PeerJ.

[38] Yue Y., Du J., Li Y., Thomas H.R., Frank M.H., Wang L., Hu H. (2021). Insight into the petunia Dof transcription factor family reveals a new regulator of male-sterility. Ind. Crop Prod.

[39] Li T., Wang X., Elango D., Zhang W., Li M., Zhang F., Pan Q., Wu Y. (2022). Genome-wide identification, phylogenetic and expression pattern analysis of Dof transcription factors in blueberry (*Vaccinium corymbosum* L.). PeerJ.

[40] Liu H., Lyu H.M., Zhu K., Van de Peer Y., Max C.Z. (2021). The emergence and evolution of intron-poor and intronless genes in intron-rich plant gene families. Plant J..

[41] Tabassum J., Raza Q., Riaz A., Ahmad S., Rashid M., Javed M.A., Ali Z., Kang F., Khan I.A., Atif R.M. (2022). Exploration of the genomic atlas of Dof transcription factor family across genus Oryza provides novel insights on rice breeding in changing climate. Front. Plant Sci..

[42] Nan H., Ludlow R.A., Lu M., An H. (2021). Genome-wide analysis of Dof genes and their response to abiotic stress in rose (*Rosa chinensis*). Front. Genet..

[43] Li J., Zhang Y., Xu L., Wang C., Luo Y., Feng S., Yuan Y., Yang Q., Feng B. (2022). Genome-wide identification of dna binding with one finger (Dof) gene family in Tartary Buckwheat (*Fagopyrum tataricum*) and analysis of its expression pattern after exogenous hormone stimulation. Biology.

[44] Meng X., Liu S., Zhang C., He J., Ma D., Wang X., Dong T., Guo F., Cai J., Long T. (2023). The unique sweet potato NAC transcription factor IbNAC3 modulates combined salt and drought stresses. Plant Physiol..

[45] Wu Z., Cheng J., Cui J., Xu X., Liang G., Luo X., Chen X., Tang X., Hu K., Qin C. (2016). Genome-wide identification and expression profile of dof transcription factor gene family in pepper (*Capsicum annuum* L.). Front. Plant Sci..

[46] Wen C.L., Cheng Q., Zhao L., Mao A., Yang J., Yu S., Weng Y., Xu Y. (2016). Identification and characterisation of Dof transcription factors in the cucumber genome. Sci. Rep..

[47] Gao H., Song W., Severing E., Vayssieres A., Huettel B., Franzen R., Richter R., Chai J., Coupland G. (2022). PIF4 enhances DNA binding of CDF2 to co-regulate target gene expression and promote Arabidopsis hypocotyl cell elongation. Nat. Plants.

[48] Santopolo S., Boccaccini A., Lorrai R., Ruta V., Capauto D., Minutello E., Serino G., Costantino P., Vittorioso P. (2015). Dof affecting germination 2 is a positive regulator of light-mediated seed germination and is repressed by dof affecting germination 1. BMC Plant Biol..

[49] Huang X., Hu L., Kong W., Yang C., Xi W. (2022). Red light-transmittance bagging promotes carotenoid accumulation of grapefruit during ripening. Commun. Biol..

[50] Yang L., Zhou S., Hou Y., Ji B., Pei L., Su X., Zhong H., Dong C. (2022). Blue light induces biosynthesis of flavonoids in *Epimedium sagittatum* (Sieb.et Zucc.) Maxim. leaves, a study on a light-demanding medicinal shade herb. Ind. Crop. Prod..

[51] Nam T.G., Kim D.O., Eom S.H. (2018). Effects of light sources on major flavonoids and antioxidant activity in common buckwheat sprouts. Food Sci. Biotechnol..

[52] Zhang S., Ma J., Zou H., Zhang L., Li S., Wang Y. (2020). The combination of blue and red LED light improves growth and phenolic acid contents in *Salvia miltiorrhiza* Bunge. Ind. Crop. Prod..

