# Supplementary material for: Identification of the Dof Gene Family in Quinoa and Its Potential Role in Regulating Flavonoid Synthesis Under Different Stress Conditions
Source: Biology (Basel). 2025 Apr 20;14(4):446. doi: 10.3390/biology14040446 (PMC12024598; doi:10.3390/biology14040446)
Supplement: Supplementary file 1 [file biology-14-00446-s001.zip › biology-3507234-supplementary.pdf]

Identification of the *Dof* Gene Family in Quinoa and Its Potential Role in Regulating Flavonoid Synthesis Under Different Stress Conditions

Guangtao Qian [1,2,3,†], Jinrong Yang [1,2,†], Mingyu Wang [3] and Lixin Li [3,*]

1 Interdisciplinary Eye Research Institute (EYE-X Institute), Bengbu Medical University, Bengbu 233030, China; qgt@bbmu.edu.cn (G.Q.); 15156172852@163.com (J.Y.)
2 School of Life Sciences, Anhui Provincial Key Laboratory of Tumor Evolution and Intelligent Diagnosis and Treatment, Bengbu Medical University, Bengbu 233030, China
3 Key Laboratory of Saline-Alkali Vegetation Ecology Restoration, Ministry of Education, School of Life Sciences, Northeast Forestry University, Harbin 150040, China; wmy19970825@163.com
* Correspondence: lixinli0515@nefu.edu.cn
† These authors contributed equally to this work.

Supplementary Table 1. All primer sequences used in this study

Gene Name	Sequence (5'-3')
qPCR-UBQ9-F	CAATGTCTACTACAACGAAGCG
qPCR-UBQ9-R	CAGACTGACCAAACACAAAGTT
qPCR-CqDof3-F	AGCGGTCACGTACTTCTTC
qPCR-CqDof3-R	ACGTAAACCCACCACCAAC
qPCR-CqDof4-F	GTGCCCTCGTTGTGATTCC
qPCR-CqDof4-R	TTTTACGGCAACCGCCTCC
qPCR-CqDof6-F	ATCTGAGGTGTCCGAGATGC
qPCR-CqDof6-R	CGCTCACCGTTGTGCTCTT
qPCR-CqDof14-F	CTACCACCTCGGCCATTGC
qPCR-CqDof14-R	ACCCACCCCTAGTCCAATACC
qPCR-CqDof21-F	CTCCGTAACGTCCCCATCG
qPCR-CqDof21-R	CCACCCAGCTGAAACTCCAT
